# Uncovering Cortical Units of Processing From Multi-Layered Connectomes

**DOI:** 10.3389/fnins.2022.836259

**Published:** 2022-03-10

**Authors:** Kristoffer Jon Albers, Matthew G. Liptrot, Karen Sandø Ambrosen, Rasmus Røge, Tue Herlau, Kasper Winther Andersen, Hartwig R. Siebner, Lars Kai Hansen, Tim B. Dyrby, Kristoffer H. Madsen, Mikkel N. Schmidt, Morten Mørup

**Affiliations:** ^1^Department of Applied Mathematics and Computer Science, Technical University of Denmark, Lyngby, Denmark; ^2^Danish Research Centre for Magnetic Resonance, Centre for Functional and Diagnostic Imaging and Research, Copenhagen University Hospital - Amager and Hvidovre, Copenhagen, Denmark; ^3^Department of Neurology, Copenhagen University Hospital Bispebjerg and Frederiksberg, Copenhagen, Denmark; ^4^Department of Clinical Medicine, Faculty of Health and Medical Sciences, University of Copenhagen, Copenhagen, Denmark

**Keywords:** multi-layered connectomes, dMRI, fMRI, stochastic block model, brain parcellation

## Abstract

Modern diffusion and functional magnetic resonance imaging (dMRI/fMRI) provide non-invasive high-resolution images from which multi-layered networks of whole-brain structural and functional connectivity can be derived. Unfortunately, the lack of observed correspondence between the connectivity profiles of the two modalities challenges the understanding of the relationship between the functional and structural connectome. Rather than focusing on correspondence at the level of connections we presently investigate correspondence in terms of modular organization according to shared canonical processing units. We use a stochastic block-model (SBM) as a data-driven approach for clustering high-resolution multi-layer whole-brain connectivity networks and use prediction to quantify the extent to which a given clustering accounts for the connectome within a modality. The employed SBM assumes a single underlying parcellation exists across modalities whilst permitting each modality to possess an independent connectivity structure between parcels thereby imposing concurrent functional and structural units but different structural and functional connectivity profiles. We contrast the joint processing units to their modality specific counterparts and find that even though data-driven structural and functional parcellations exhibit substantial differences, attributed to modality specific biases, the joint model is able to achieve a consensus representation that well accounts for both the functional and structural connectome providing improved representations of functional connectivity compared to using functional data alone. This implies that a representation persists in the consensus model that is shared by the individual modalities. We find additional support for this viewpoint when the anatomical correspondence between modalities is removed from the joint modeling. The resultant drop in predictive performance is in general substantial, confirming that the anatomical correspondence of processing units is indeed present between the two modalities. Our findings illustrate how multi-modal integration admits consensus representations well-characterizing each individual modality despite their biases and points to the importance of multi-layered connectomes as providing supplementary information regarding the brain's canonical processing units.

## 1. Introduction

The prominent approach of viewing the organization of the brain at the macro scale needs to reconcile two fundamental aspects: while the cortex is *segregated* into specialized neuronal regions, the cognitive functions emerge from *integration* of these regions by coordinated activation (Tononi et al., 1994). Network science provides a convenient way to model complex relational systems, such as the behavior of the human brain, which does not emerge solely from the properties of the individual units, but from the complex interactions between these. Here, both aspects of brain organization can be summarized as networks, reflecting the *structural* and *functional* connectivity respectively, thereby permitting network science to provide the statistical foundation and methodology for investigating and quantifying the organization of brain connectivity (Bullmore and Sporns, 2009; Van Den Heuvel and Pol, 2010). Recent proposals aim at jointly modeling multiple modalities of brain connectivity using multi-layer networks (Battiston et al., 2017; Buldú and Porter, 2017; De Domenico, 2017), where the connections from different modalities are encoded within different layers, sharing the same network nodes (Betzel and Bassett, 2016), see also Vaiana and Muldoon (2020) for a recent review. Such multi-layer investigations allow neuroscience to integrate the complementary aspects of structural and functional data. However, the implications of multimodal integration, the extent to which it is interpretable, and the correspondence between the modalities remain unclear (Battiston et al., 2017; De Domenico, 2017).

Direct comparisons of structural and functional connectivity derived from diffusion and functional magnetic resonance imaging (dMRI/fMRI) have shown that structure to some degree reflects function (Koch et al., 2002; Greicius et al., 2009; Sporns, 2014). This suggests that a relationship does exist between the two modalities, indicated by measures of network properties, e.g., functional connectivity networks exhibiting various small-world attributes (Achard et al., 2006), which could be reflected by an evolutionarily-sound and economically-efficient structure (Bullmore and Sporns, 2009). However, the time scales of structural and functional connectivity derived from MRI are orders of magnitude apart. As such, the blood-oxygen-level-dependent (BOLD) hemodynamics quantified by fMRI are in the order of seconds with observed responses to stimuli delayed by at least a second and peaking after 4–8 s (Kim and Bandettini, 2012). Structural connections on the other hand operate in the order of milliseconds (Innocenti et al., 2014) which can thus not be directly probed by fMRI. Notably, the low temporal resolution of fMRI can be overcome by other functional neuroimaging methods such as electroencephalography (Deslauriers-Gauthier et al., 2019) but at the cost of low spatial resolution. At the whole-brain scale, previous studies suggest that functional connectivity quantified by fMRI to some extent emerges from the structural organization (Greicius et al., 2009; Sporns, 2014; Becker et al., 2015), but BOLD derived functional connectivity has also been observed between cortical regions that are not directly anatomically connected (Koch et al., 2002; Vincent et al., 2007; Skudlarski et al., 2008; Honey et al., 2009). In particular, stronger prevalence of functional connections linking right and left hemispheres have been observed (Koch et al., 2002; Vincent et al., 2007; Skudlarski et al., 2008). Additionally, various neurological disorders have been shown to cause alterations in both functional and structural connectivity (Fornito and Bullmore, 2012; Tost et al., 2012; Kaiser, 2013; van Dellen et al., 2013), though the extent to which any relation between functional and structural connectivity affects brain disease still needs further investigation (Vega Pons et al., 2016). Thus, although BOLD functional connectivity to some extent has been found to correlate with the strength of the direct anatomical connections as quantified by the number of streamlines between regions (Honey et al., 2009; Hermundstad et al., 2013), structural and BOLD functional connectivity operate on vastly different time-scales. As a consequence, the direct structural connections are not found to be very predictive of functional connections but moreso when integrating multiple steps in the structural connectome (Røge et al., 2017).

Existing attempts at jointly modeling functional and structural connectomes have primarily focused on how structure can inform function (Hinne et al., 2014) or function enhance recovery of structural connections (Chu et al., 2018). In Zhang et al. (2021), canonical correlation analysis (CCA) was used to identify optimal projections maximizing the correlation between structural and functional connections, and in Becker et al. (2018) spectral methods were used to relate structural connections and paths along the structural graph to functional connectivity. Structural and functional connectomes have further been jointly modeled using independent component analysis (ICA) combining structural and functional connections as features for the ICA (Amico and Goñi, 2018). Recently, deep learning autoencoders (Banka et al., 2020) and graph neural networks (see also Bessadok et al., 2021 for an overview) have been proposed for multimodal integration providing non-linear mappings between structural and functional connectivity (Li et al., 2021) as well as joint learning of connectivity fingerprints predictive of phenotypic traits (Filip et al., 2020; Dsouza et al., 2021). In the context of connectivity based parcellations the most prominent approaches have been to use k-means, hierarchical, or spectral clustering (Eickhoff et al., 2015; Reuter et al., 2020) to parcellate functional and structural connectivity. Whereas these frameworks can provide joint parcellations as a post processing step, joint parcellations using generic heterogeneous data clustering tools (Liu et al., 2020) based on Gabasova et al. (2017) have also been considered.

In this article, we approach the assessment of concurrence between functional connectivity (FC) and structural connectivity (SC) of high-resolution multi-layered connectomes, *hypothesizing that correspondence occurs not at the level of connectivity but organization of latent processing units*. We therefore assume that both the structural and functional connectome express different connectional fingerprints of the brain's canonical processing units. This assumption is formalized in the multi-layered network by assuming that measurements from differing modalities (i.e., network-layers) originate from the same processing units (i.e., group of network nodes) but with substantial differences in how the connectivity profiles between these processing units are expressed across modalities. As a result, even though the elicited networks of SC and FC are different, we hypothesize that they both reflect the same underlying organization, that would emerge if neurobiological *atoms* of the cortex were to form aggregated regions which are shared across both modalities (Eickhoff et al., 2017). In particular, focusing on the consensus representation obtained by combining modalities may better reveal these regions, by providing a representation that is less polluted by modality specific biases.

**Figure 1:**
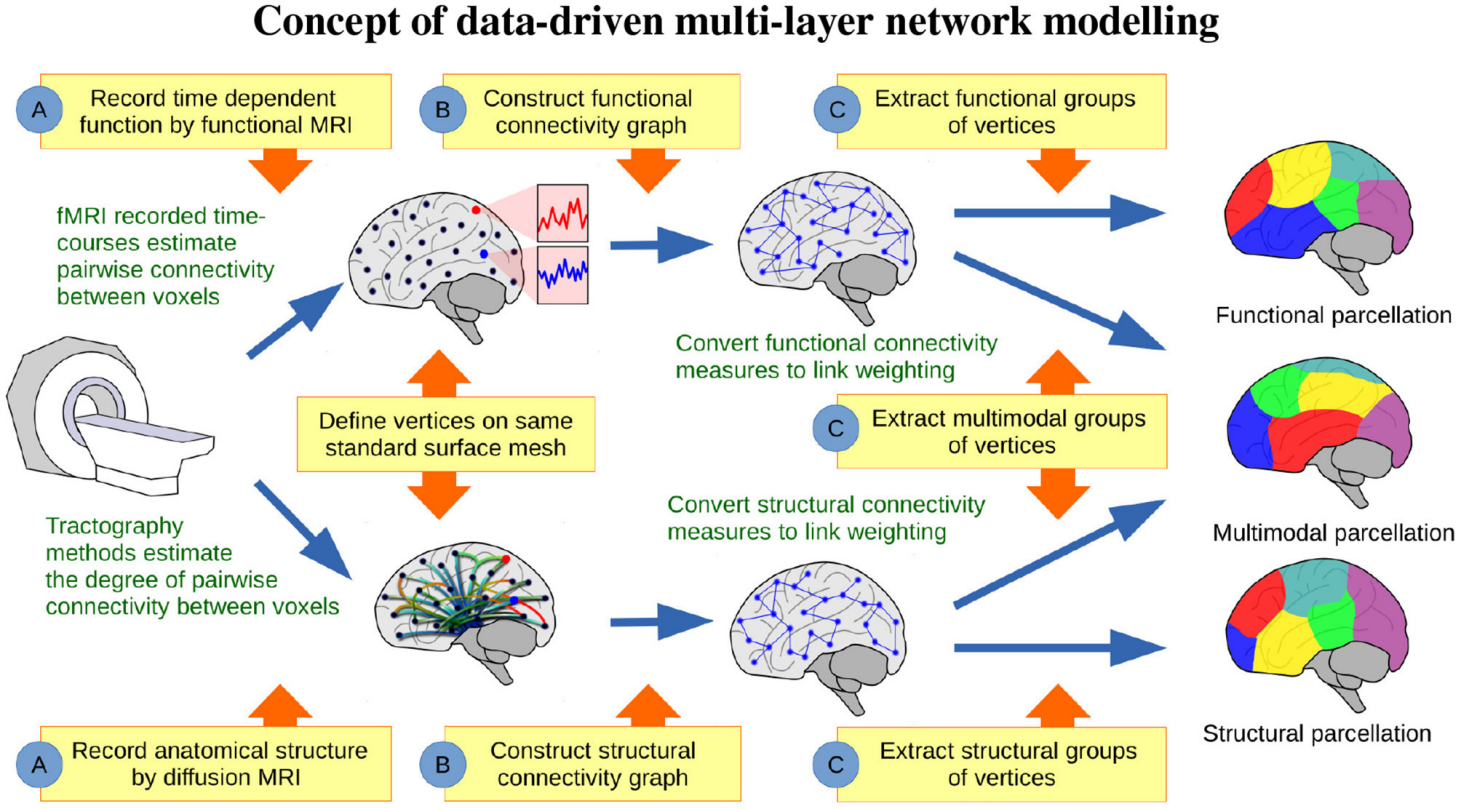
Concept of data-driven multi-layer network modeling. **(A)** Based on functional and structural MRI, graphs of structural and functional connectivity are generated, **(B)** such that vertices are defined on the same standard surface mesh. **(C)** From these graphs data-driven parcellations can be inferred and compared, when modeling structure or function either individually or jointly.

**Figure 2:**
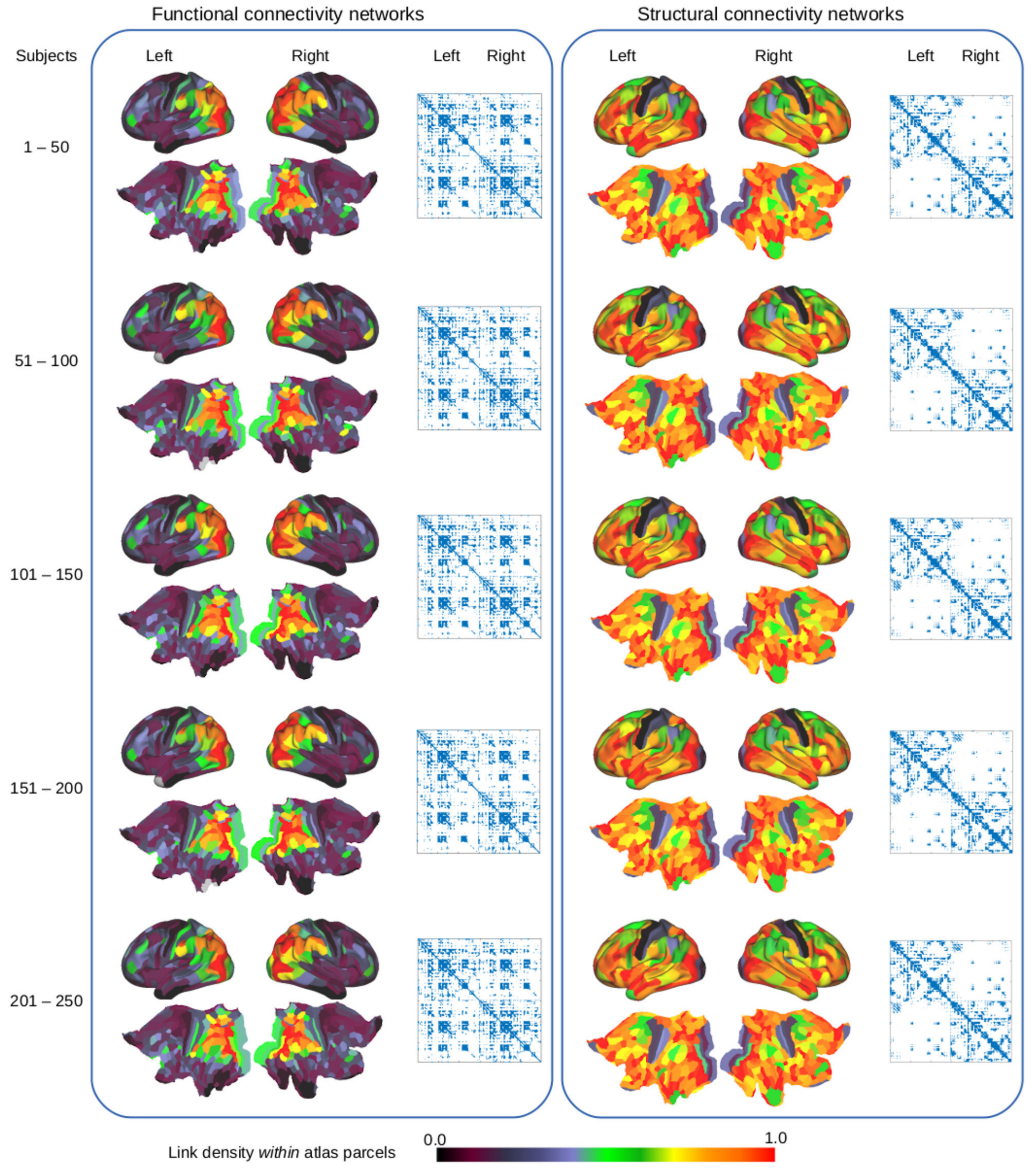
Examination of functional connectivity **(left)** and structural connectivity **(right)** using the HCP_MMP1.0 atlas parcellation (Glasser et al., 2016). The cortical surface and flatmaps show the link-density within each of the 360 atlas parcels whereas the adjacency matrices outline the whole brain functional and structural connectivity graphs, based on the average of different populations of 50 subjects.

Figure 1 illustrates the conceptual steps of the data-driven approach for exploring the organization of the brain, based on network modeling of structural and functional neuroimaging data. We presently consider data obtained from the publicly released Human Connectome Project (HCP) (Feinberg et al., 2010; Moeller et al., 2010; Setsompop et al., 2012; Xu et al., 2012; Van Essen et al., 2013) database, from which we generated *in vivo* whole-brain resting state FC and SC networks for a large population of 250 healthy subjects. The networks were inferred from functional and diffusion recordings in the full image resolution supported by modern MRI. The graphs for each subject were binarized and thresholded at one percent density, ignoring the sub-cortical voxels, and hence each contain 59,412 vertices. These graphs were then randomly split into five populations of 50 subjects, aggregated into single functional and structural networks for each group, and finally binarized to one percent link-density. Figure 2 illustrates how the profiles of structural and functional connectivity substantially differ both in terms of strength within anatomical regions and in their whole brain connectional fingerprint, whilst relatively exhibiting limited variation within each modality across populations of 50 subjects. In particular, functional connectivity shows a high degree of inter-hemispheric connections when compared to the structural connectivity that is mainly ipsi-lateral. Apart from time-scale differences, the lack of inter-hemispheric structural connections can be attributed to limitations of current tractography methods (Maier-Hein et al., 2017). As such, the average area under curve (AUC) of the receiver operator characteristic directly predicting the connectivity of one group of subjects from another group of subjects (i.e., considering the total number of 0–0 matches, 0–1 matches, 1–0 matches, and 1–1 matches; Ambrosen et al., 2014; Røge et al., 2017) across the FC graphs is 0.901 whereas it is 0.935 for the SC graphs and 0.618 predicting FC from SC for the same group of subjects.

This lack of concurrence at the modality-specific connectivity level both within and between parcels does not rule out concurrence at the level of the underlying processing units. If the processing units resolved by both modalities are in perfect agreement, the inter-population variability of these units within each modality would be comparable to their inter-modality variability within populations. However, observed differences in network properties can be due to differing sources, such as noise in the data and measurement procedure including scanning parameters (Ambrosen et al., 2020), as well as inherent differences in the signals measured by the modalities including time scales as discussed above. For example, fMRI is known to suffer from motion artifacts (Diedrichsen and Shadmehr, 2005), whereas diffusion MRI is known to exhibit biases such as preference of tractography methods to terminate at gyral crowns (Schilling et al., 2018). We thus expect that modality specific biases are present and that they will drive parcellations in disagreeing directions. To investigate this we provide both a qualitative characterization as well as a quantitative predictive assessment of the differences of data driven structural and functional parcellations and contrast this to the consensus representation obtained by joint modeling of the structural and functional connectome.

**Figure 3:**
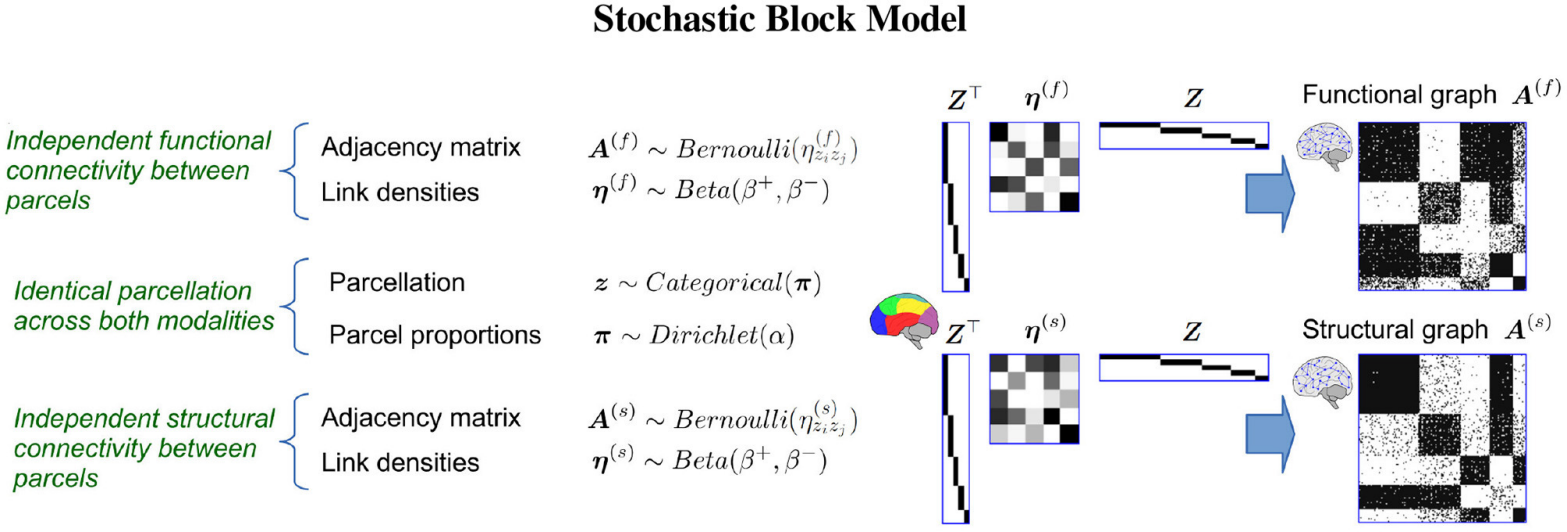
The Stochastic Block Model (SBM) is a generative model capable of discovering a single group-structure from multiple complex networks, i.e., functional connectome ***A***^(*f*)^ and structural connectome ***A***^(*s*)^. Based on such a shared parcellation ***z***, the model assumes links are independently generated from a Bernoulli distribution such that the probability of observing a link between any two vertices only depends on the modality specific probability of observing a link between the two parcels that the vertices belong to given by the inter parcel (off diagonal elements) and intra parcel (diagonal elements) of the link density matrices **η**^(*f*)^ and **η**^(*s*)^ for the functional and structural connectomes, respectively. Under this assumption, the model hence allows a single parcellation to be inferred from multiple networks while accounting for differences in connectivity profiles. Let ***Z*** denote a matrix of the clustering ***z*** using a one-hot encoding. Notably, the SBM can be considered a lossy compressed representation of the connectomes such that ***A***^(*f*)^ ≈ ***Z***^⊤^**η**^(*f*)^***Z*** and ***A***^(*s*)^ ≈ ***Z***^⊤^**η**^(*s*)^***Z***.

**Figure 4:**
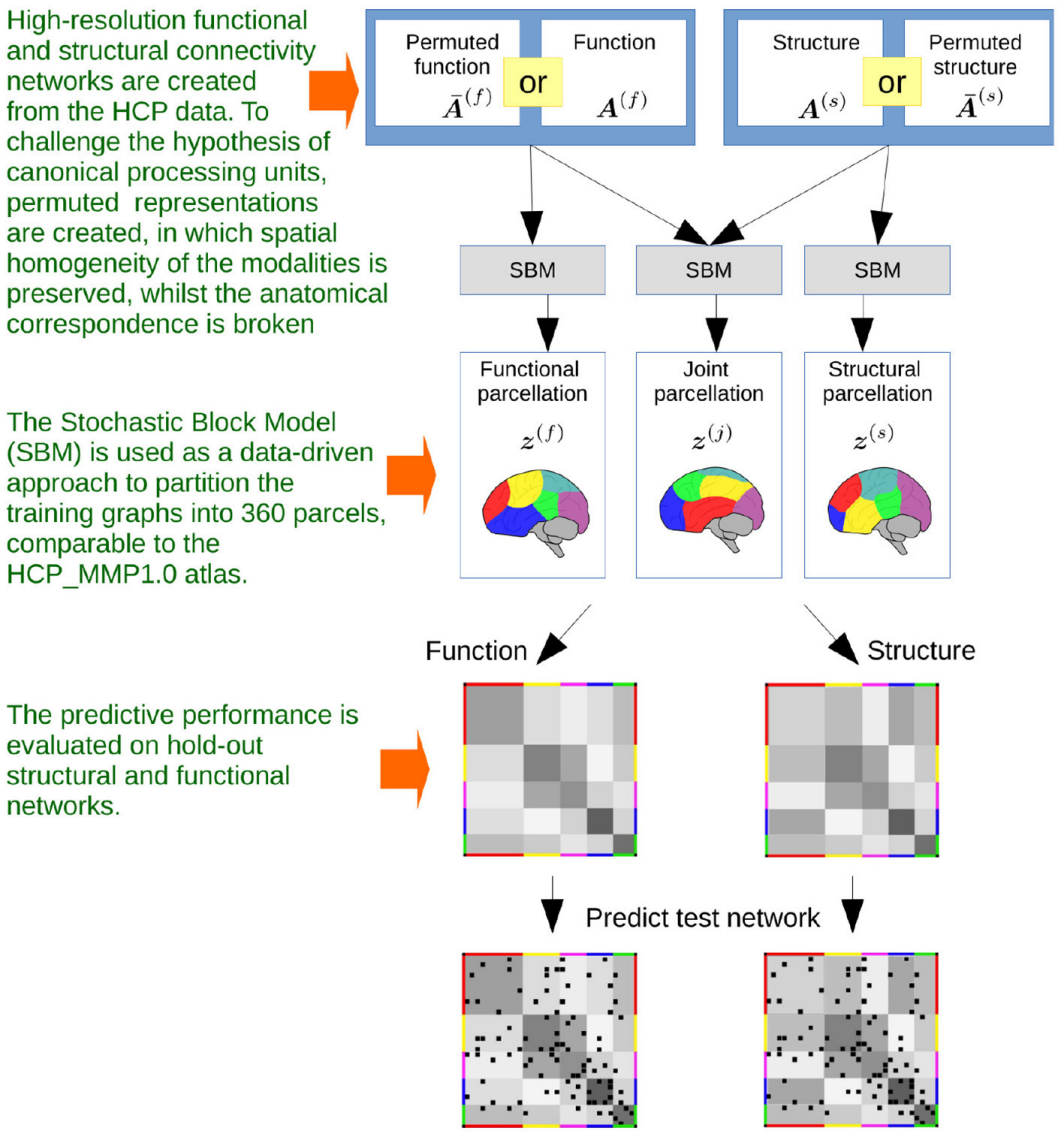
The steps that defines the flow of the investigations. Based on high-resolution functional and structural MRI obtained from publicly released data of the Human Connectome Project, independent networks of structural and functional connectivity are generated. The networks are based on population averages, resulting in a total of five networks for each modality, based on five populations of 50 subjects. The networks are binarized by thresholding at 1% link density. Stochastic Blockmodeling is utilized to infer data-driven parcellations, based on modeling structure or function individually or jointly modeling both modalities for the five populations. The benefits of multimodal integration are hence evaluated by comparing the performance of predicting hold-out population networks using inferred single and multimodal parcellations, contrasted with that of using networks that have been spatially permuted whilst preserving the size of the parcels.

We use a stochastic block model (SBM) (Nowicki and Snijders, 2001) which allows us to infer a single parcellation based upon multiple networks (see Figure 3). An SBM type of framework has previously been used for functional (Mørup et al., 2010; Andersen et al., 2014; Baldassano et al., 2015) and structural parcellation (Ambrosen et al., 2014; Baldassano et al., 2015) as well as joint modeling of functional and structural connectivity in low resolution (116 network nodes; Andersen et al., 2012a). We provide statistical evaluation of the predictive performance of the inferred parcellations following a similar framework to the one proposed in Albers et al. (2021) (see Figure 4). We compare the results of joint modeling with the comprehensive HCP_MMP1.0 atlas which is constructed using multiple modalities, including neuroanatomy (Glasser et al., 2016). We further contrast the results to a non-trivial (block permuted) null hypothesis of *non-correspondence* between the structural and functional regions. We exploit that the HCP vertex order is spatially contiguous and that a simple permutation in which the non-predicted modality is permuted according to a parcellation learned on the modality thereby preserves spatial contiguous network blocks with similar size distribution in both modalities while breaking any anatomical correspondence. In summary, we test the hypothesis that structural and functional connectomes derived from dMRI and fMRI support shared canonical processing units by:

(A) Characterizing differences of modality specific and multi-modal data-driven parcellations.
(B) Contrasting the predictive performance of parcellations trained on the same modality, different modality, or trained jointly using both modalities.
(C) Contrasting the predictive performance to the performance obtained when permuting the connectome of one modality thereby enforcing non-correspondence.

The novelty of this work lies both in the characterization of the multimodal concurrence identified in the high-resolution HCP data, despite the substantial differences in the observed connectivity profiles, and in the application of a quantitative predictive framework to assessing the validity of canonical processing units.

## 2. Materials and Methods

### 2.1. Data

Magnetic Resonance Imaging (MRI) techniques provide non-invasive means from which functional and structural connectivity networks can be constructed. Structural connectivity can be derived from diffusion MRI (Gong et al., 2009) by tracking white matter streamlines across the cortex such that structural networks are obtained based on the anatomy of the brain. Functional MRI captures images of functional whole brain connectivity by indirectly measuring the time-dependent neural activity within small regions of the brain (i.e., voxels) by monitoring the blood oxygenation level dependent (BOLD) response (Ogawa et al., 1990). Networks of functional connectivity can be obtained, for instance as mapped by the correlated activation of brain regions (Bullmore and Sporns, 2009).

Networks of functional and structural brain connectivity were obtained using independent high-resolution data from the Human Connectome Project (HCP) (Feinberg et al., 2010; Moeller et al., 2010; Setsompop et al., 2012; Xu et al., 2012; Van Essen et al., 2013) database available from the MGH-USC Human Connectome Project (HCP) database (https://ida.loni.usc.edu/login.jsp). Ignoring the sub-cortical information, the networks contained 59,412 vertices covering the neocortex. We split the 250 subjects into populations, such that we obtained five non-overlapping groups of 50 subjects. For each group we created a single functional and structural training network based on the group average. The MRI data for all the subjects in each population were aggregated before the networks were constructed and thresholded in order to obtain a single functional and structural network representative for the group.

The fMRI networks were estimated from the preprocessed and structurally denoised ICA-FIX cleaned version of the resting state fMRI data, for further reference see Smith et al. (2013), Griffanti et al. (2014), and Salimi-Khorshidi et al. (2014). We formed the networks by averaging the Pearson correlation matrix estimated from the two sessions using both the left-right and right-left phase encoding directions for each subject (i.e., averaging four correlograms per subject each estimated from 1,200 time frames).

The structural connectivity networks were derived from the dMRI data preprocessed using the HCP pipeline (Glasser et al., 2013). The fiber orientation estimation was done using FSL's BedpostX for multi-shell data (Jbabdi et al., 2012) and the networks were constructed by performing probabilistic tractography using FSL's Probtrackx2 (Behrens et al., 2003, 2007) run in “matrix3” mode. One thousand streamlines were initiated in each white matter voxel, and a resulting streamline was kept if it reached two vertices of the white matter surface, resulting in weighted graphs of streamline counts between vertices. The adjacency matrices for all subjects in the group were added and binarized by thresholding the graph at 1% density keeping only the strongest links.

### 2.2. The HCP_MMP1.0 Atlas

To ground results, we contrasted the performance obtained using stochastic block modeling to the performance using a prominent existing parcellation, i.e., the HCP_MMP1.0 (Glasser et al., 2016) atlas. The HCP_MMP1.0 atlas (Glasser et al., 2016) is based on multi-modal MRI data from the HCP and describes a total of 360 parcels split equally across both hemispheres. It was created in a combined data-driven and manual approach to obtain a single parcellation of cortical regions, based on multiple neurobiological properties including both functional information and brain anatomy obtained from 210 healthy subjects. We have previously found this atlas to perform relatively well when predicting single subject structural and functional connectivity networks and therefore include it presently as a baseline (Albers et al., 2021).

### 2.3. Joint Integration Using the Stochastic Block Model

Both in terms of its structural organization and functional activity the brain can be studied as a network. One approach of quantifying the latent structure in connectivity networks is to partition the nodes into groups that share a similar connectivity pattern within the network. The stochastic block model (SBM) (Nowicki and Snijders, 2001) is a data-driven Bayesian clustering approach, which, coupled with Markov Chain Monte Carlo (MCMC) sampling, has proven a valid tool for clustering and investigating structure in complex networks (Zhu et al., 2008; Schmidt and Mørup, 2013). Notably, a non-parametric SBM modeling framework [denoted the infinite relational model (IRM)] (Kemp et al., 2006; Xu et al., 2006) has previously been used for the separate modeling of functional (Mørup et al., 2010; Andersen et al., 2012b, 2014) and structural connectivity (Ambrosen et al., 2013, 2014) whereas joint modeling of structural and functional connectivity has been considered in Andersen et al. (2012a). Notably, the approach of Andersen et al. (2012a) was based on low resolution networks of 116 nodes defined by the AAL atlas (Tzourio-Mazoyer et al., 2002) with the ability to impose shared and individual segregated units of the two modalities.

The stochastic block model (SBM) (Nowicki and Snijders, 2001) partitions network nodes into clusters with similar connectivity patterns. For modeling symmetric binary networks, the model can be defined by the following generative process, where *m* is used to index modality:


(1) $$\begin{array}{l} \text{Links in network: }A_{ij}^{\left(m\right)}\sim \text{ Bernoulli}\left(\eta_{z_{i}z_{j}}^{\left(m\right)}\right), \end{array}$$

(2) $$\begin{array}{l} \text{Cluster-link densities: }\eta_{\ell h}^{\left(m\right)}\sim \text{ Beta}\left(\beta^{+},\beta^{-}\right), \end{array}$$

(3) $$\begin{array}{l} \text{Clustering: }z_{i}\sim \text{ Categorical}\left(\pi \right), \end{array}$$

(4) $$\begin{array}{l} \text{Cluster proportions: }\pi \sim \text{ Dirichlet}\left(\alpha \right). \end{array}$$

The probability of observing a link between two nodes *i* and *j* in the network follows a Bernoulli distribution only depending on the probability of observing links between the clusters *z*_*i*_ and *z*_*j*_ that the nodes belong to. The probability of observing links between two clusters is considered independent given the assignment to clusters and follows a Beta distribution. Finally, the nodes are partitioned into *K* clusters, and the cluster proportions follow a Dirichlet distribution.

The stochastic block model when used for multimodal integration is outlined in Figure 3. The observed functional and structural connectomes ***A***^(*f*)^ and ***A***^(*s*)^ are assumed to be generated according to a shared functional and structural parcellation ***z*** such that *z*_*i*_ = ℓ indicates that vertex *i* belongs to parcel ℓ. Whereas, the parcellation is shared, the manner in which the different regions integrate is assumed to be modality specific and parameterized respectively for the functional and structural connectomes by $\eta_{\ell h}^{\left(f\right)}$ and $\eta_{\ell h}^{\left(s\right)}$ providing the extent (i.e., the probability) that nodes in parcel ℓ connect to nodes in parcel *h*. As a result, the observed connectomes can have substantially different within and between parcel connectivity structures ***η***^(*f*)^ and ***η***^(*s*)^ while being defined in terms of the same underlying units of processing ***z***. As parcels may differ in size, **π** is used to account for size-heterogeneity.

Due to the conjugacy between the Dirichlet and Categorical distribution, **π** can be analytically marginalized (see Schmidt and Mørup, 2013 for details). By imposing an equal concentration parameter for all *K* clusters $\alpha =\frac{\alpha }{K}1_{K\times 1}$ the following effective prior for the clustering can be obtained:


(5) $$P(z|\alpha )=\frac{\Gamma (\alpha )}{\Gamma (\alpha +N)}\prod_{l=1}^{K}\frac{\Gamma (\frac{\alpha }{K}+n_{k})}{\Gamma (\frac{\alpha }{K})},$$

where *N* is the number of nodes, *n*_*k*_ is the number of nodes in cluster *k*, and Γ(*x*) is the gamma function. Notably, we use the SBM to obtain a *single* parcellation based on either a single network from one modality (either functional or structural) or two networks, one for each modality when jointly modeling structure and function. Let ***A*** represent the set of *M* networks, containing either *M* = 1 or *M* = 2 modalities. The beta prior is conjugate to the Bernoulli likelihood, which allows us to obtain the following joint distribution as ***η*** can be analytically marginalized:


(6) $$\begin{array}{l} P\left(A,z|\beta^{+},\beta^{-},\alpha \right)=P\left(z|\alpha \right)\overset{M}{\underset{m}{\prod }}\underset{\ell \leq h}{\prod }\frac{B\left(N_{\ell h}^{\left(m\right)+}+\beta^{+},N_{\ell h}^{\left(m\right)-}+\beta^{-}\right)}{B\left(\beta^{+},\beta^{-}\right)}, \end{array}$$

where $N_{\ell h}^{\left(m\right)+}=\underset{1\leq i<j\leq N}{\sum }\delta_{z_{i},\ell }\delta_{z_{j},h}A_{ij}^{\left(m\right)}$ and $N_{\ell h}^{\left(m\right)-}=\underset{1\leq i<j\leq N}{\sum }\delta_{z_{i},\ell }\delta_{z_{j},h}\left(1-A_{ij}^{\left(m\right)}\right)$ respectively represent the number of links and non-links between cluster ℓ and *h* according to network ***A***^(*m*)^, while $B\left(a,b\right)=\frac{\Gamma \left(a\right)\Gamma \left(b\right)}{\Gamma \left(a+b\right)}$ is the beta function.

### 2.4. Model Inference

We infer the model parameters using a Markov Chain Monte Carlo (MCMC) procedure. The parcellation is inferred by Gibbs sampling, where the assignment *z*_*i*_ for each node *i* in turn is processed, based on the posterior distribution for the assignment of *i* to each of the *K* clusters ℓ. Using Bayes' theorem this can be obtained from Equation 6, where $z^{\backslash i}={\left(z_{j}\right)}_{j\neq i}$ are the cluster assignments for all nodes ignoring node *i*:


(7) $$\begin{array}{l} P\left(z_{i}=\ell |A,z^{\backslash i},\beta^{+},\beta^{-},\alpha \right)=\frac{P\left(A,z^{\backslash i},z_{i}=\ell |\beta^{+},\beta^{-},\alpha \right)}{\overset{K}{\underset{h=1}{\sum }}P\left(A,z^{\backslash i},z_{i}=h|\beta^{+},\beta^{-},\alpha \right)}. \end{array}$$

For inferring the hyper-parameters, β^+^, β^−^, and α, we use a simple Metropolis-Hastings procedure, where new proposals are drawn from a Gaussian distribution centered at the current parameter value with variance 1.

For all experiments the model parameters are inferred by sampling 100 iterations of the following sampling procedure: ***z*** is updated by one complete Gibbs sweep over all nodes followed by 1,000 MH-proposals for updating each hyper-parameter β^+^, β^−^, α. Due to the size of the networks, it is not computationally feasible to reach convergence. However, the Gibbs sampler quickly reach a stable cluster assignment with high posterior likelihood which is treated as a point estimate of the parameters (Albers et al., 2013). We hence treat the last sampled state as the inferred parameters. All experiments are performed with *K* = 360 clusters which limits SBM to the same complexity as the HCP_MMP1.0 atlas. For the HCP_MMP1.0 the hyperparameters, β^+^ and β^−^, are inferred for each of the training networks, using the Metropolis-Hastings procedure with the parcellation fixed to the HCP atlas. Code for the SBM modeling framework is provided at brainconnectivity.compute.dtu.dk.

### 2.5. Predictive Performance

To assess and compare the quality of parcellations we use the predictive framework established in Ambrosen et al. (2013) and Albers et al. (2021). The quality of a parcellation is evaluated by how well it can be used to predict unseen held-out networks.

#### 2.5.1. Predictive Likelihood

Let ***A***^(*m*)*train*^ denote the training network and ***A***^(*m*)*test*^ the network used for evaluating the learned parcellation ***z*** and how these segregated units integrate in terms of their intra and inter connectivity densities defined by the matrix ***η***^(*m*)^. The expected predictive log-likelihood is then given by:


(8) $$\begin{array}{c} {\langle \log p\left(A^{\left(m\right),test}|A^{\left(m\right),train},z,\beta^{+},\beta^{-},\alpha \right)\rangle }_{p\left(\eta^{\left(m\right)}|A^{\left(m\right),train},z\right)}=\\ \;\underset{i>j}{\sum }A_{ij}^{\left(m\right),test}{\langle +\log\left(\eta_{z_{i}z_{j}}^{\left(m\right)}\right)\rangle }_{p\left(\eta^{\left(m\right)}|A^{\left(m\right),train},z\right)}\\ \;\left(1-A_{ij}^{\left(m\right),test}\right){\langle \log\left(1-\eta_{z_{i}z_{j}}\right)\rangle }_{p\left(\eta^{\left(m\right)}|A^{\left(m\right),train},z\right)}, \end{array}$$

where the expectations are given with respect to the distribution *p*(***η***^(*m*)^|***A***^(*m*), *train*^, ***z***) as:


(9) $$\begin{array}{l} {\langle \log\left(\eta_{\ell h}^{\left(m\right)}\right)\rangle }_{p\left(\eta^{\left(m\right)}|A^{\left(m\right),train},z\right)}=\psi \left(N_{\ell h}^{\left(m\right)+}+\beta^{+}\right)\\ \;-\psi \left(N_{\ell h}^{\left(m\right)+}+N_{\ell h}^{\left(m\right)-}+\beta^{+}+\beta^{-}\right) \end{array}$$

(10) $$\begin{array}{l} {\langle \log\left(1-\eta_{\ell h}^{\left(m\right)}\right)\rangle }_{p\left(\eta^{\left(m\right)}|A^{\left(m\right),train},z\right)}=\psi \left(N_{\ell h}^{\left(m\right)-}+\beta^{-}\right)\\ \;-\psi \left(N_{\ell h}^{\left(m\right)+}+N_{\ell h}^{\left(m\right)-}+\beta^{+}+\beta^{-}\right), \end{array}$$

with ψ being the digamma function $\psi \left(x\right)=\frac{d}{dx}\log\left[\Gamma \left(x\right)\right]$. In Albers et al. (2021), the log of the expected predictive likelihood was also considered but found to provide similar performance to the expected predictive log-likelihood and therefore not included herein.

#### 2.5.2. Area Under Curve

An alternative measure is to describe how the probabilities of generating links inferred by SBM from the training network can be used to separate between links and non-links in the test network. We quantify this performance by the area under curve (AUC) of the Receiver Operator Characteristics curve (ROC) (Clauset et al., 2008), scored by the expected link probability between clusters as observed from the training graph


(11) $$\begin{array}{l} \langle \eta_{z_{i}z_{j}}^{\left(m\right)}\rangle =\frac{N_{z_{i}z_{j}}^{\left(m\right)+}+\beta^{+}}{N_{z_{i}z_{j}}^{\left(m\right)+}+N_{z_{i}z_{j}}^{\left(m\right)-}+\beta^{+}+\beta^{-}}. \end{array}$$

These scores are then evaluated in terms of how well they, for the corresponding entries $A_{ij}^{\left(m\right),test}$ in the test graph, are able to separate links (considered the positive class) from non-links (considered the negative class). Let $R_{ij}^{\left(m\right),test}=\langle \eta_{z_{i}z_{j}}^{\left(m\right)}\rangle$ be the reconstructed test connectome for modality *m* and vec_U_(***B***) return the upper triangular part of the matrix ***B***^*I*×*I*^ as the vector ***b***^*I*·(*I*−1)/2×1^. The score vector $ŷ=\mathrm{ve}{\text{c}}_{\text{U}}\left[R^{\left(m\right),test}\right]$ and true labels $y=\mathrm{ve}{\text{c}}_{\text{U}}\left[A^{\left(m\right),test}\right]$ can then be considered as inputs to the standard receiver operator characteristic function for calculating the area under curve (AUC).

### 2.6. Parcellation Comparison by Mutual Information

The similarity of different parcellations can be quantified using Mutual Information (MI). This constitutes a permutation invariant measure for the shared clustering information between two parcellations **z** and **z**′ given by:


(12) $$\begin{array}{l} \text{MI}\left(z,z^{\prime }\right)=\underset{cc^{\prime }}{\sum }P\left(c,c^{\prime }\right)\log\left(\frac{P\left(c,c^{\prime }\right)}{P\left(c\right)P\left(c^{\prime }\right)}\right), \end{array}$$

where $P\left(c\right)=\overset{K}{\underset{c^{\prime }}{\sum }}P\left(c,c^{\prime }\right)$ is the probability of observing a node in cluster *c* while $P\left(c,c^{\prime }\right)=\frac{1}{N}\overset{N}{\underset{i=1}{\sum }}\delta_{z_{i},c}\delta_{z_{i}^{\prime },c^{\prime }}$ is the probability of jointly observing a node in cluster *c* in **z** and a node in cluster *c*′ in *****z***′**. We use the normalized mutual information (NMI) to get a value between zero and one:


(13) $$\begin{array}{l} \text{NMI}\left(z,z^{\prime }\right)=\frac{2\text{MI}\left(z,z^{\prime }\right)}{\text{MI}\left(z,z\right)+\text{MI}\left(z^{\prime },z^{\prime }\right)}, \end{array}$$

such that a value of one indicates that the parcellations are identical.

### 2.7. Blocked Permutation Procedure

**Figure 5:**
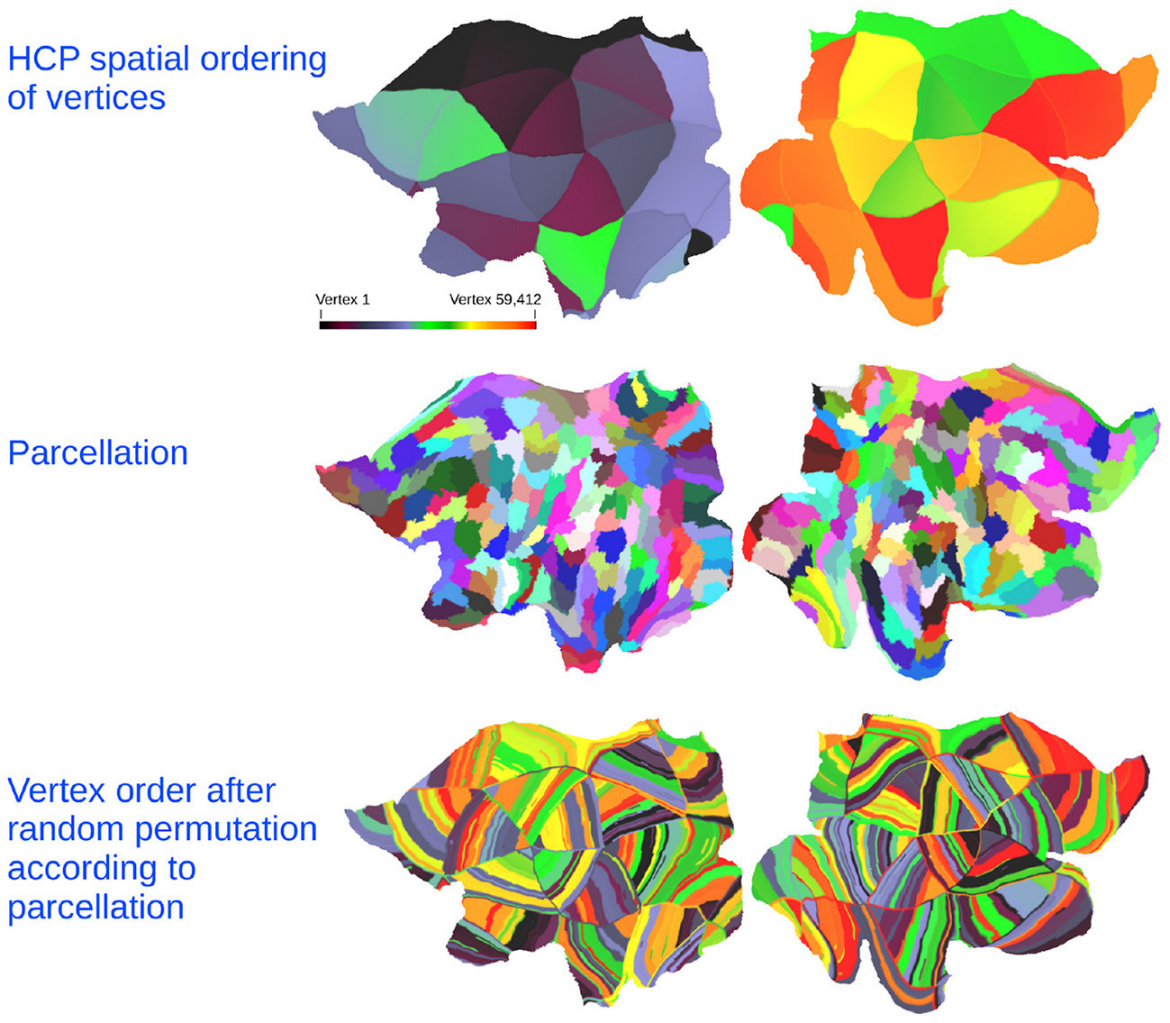
**(Top)** Flatmap of each vertex in the HCP data color-coded according to the vertex traversal order of the network adjacency matrices. **(Middle)** Example of parcellation structure learned from the non-predicted modality. **(Bottom)** The random permutation of the non-predicted modality obtained by re-ordering the vertex traversal according to the learned parcellation structure in which the clusters are ordered in random order. Color-code indicates the original vertex position.

To probe the correspondence of the extracted structural and functional units and their joint integration we contrast the performance to a null hypothesis assuming no correspondence. To achieve this, we use a permutation procedure that accounts for size distribution and to some extent for spatial contiguity, while upholding the assumption that the parcels do not correspond in the two modalities. The permutation procedure re-organizes all vertices of the non-predicted modality according to a clustering structure learned on the non-predicted modality in which clusters are ordered in random order. (That is, when predicting the functional connectome the vertices of the structural connectome are re-organized and vice versa). Thereby, the non-predicted modality is ordered in terms of modality specific units such that the vertices of these units correspond to different spatial contiguous regions defined through the HCP vertex traversal order in the predicted modality. The permutation procedure is illustrated in Figure 5. This procedure preserves size distribution and spatial contiguity as defined by the HCP vertex traversal order but does not account for anatomy nor spatial shape.

## 3. Results

### 3.1. Data-Driven Parcellations

**Figure 6:**
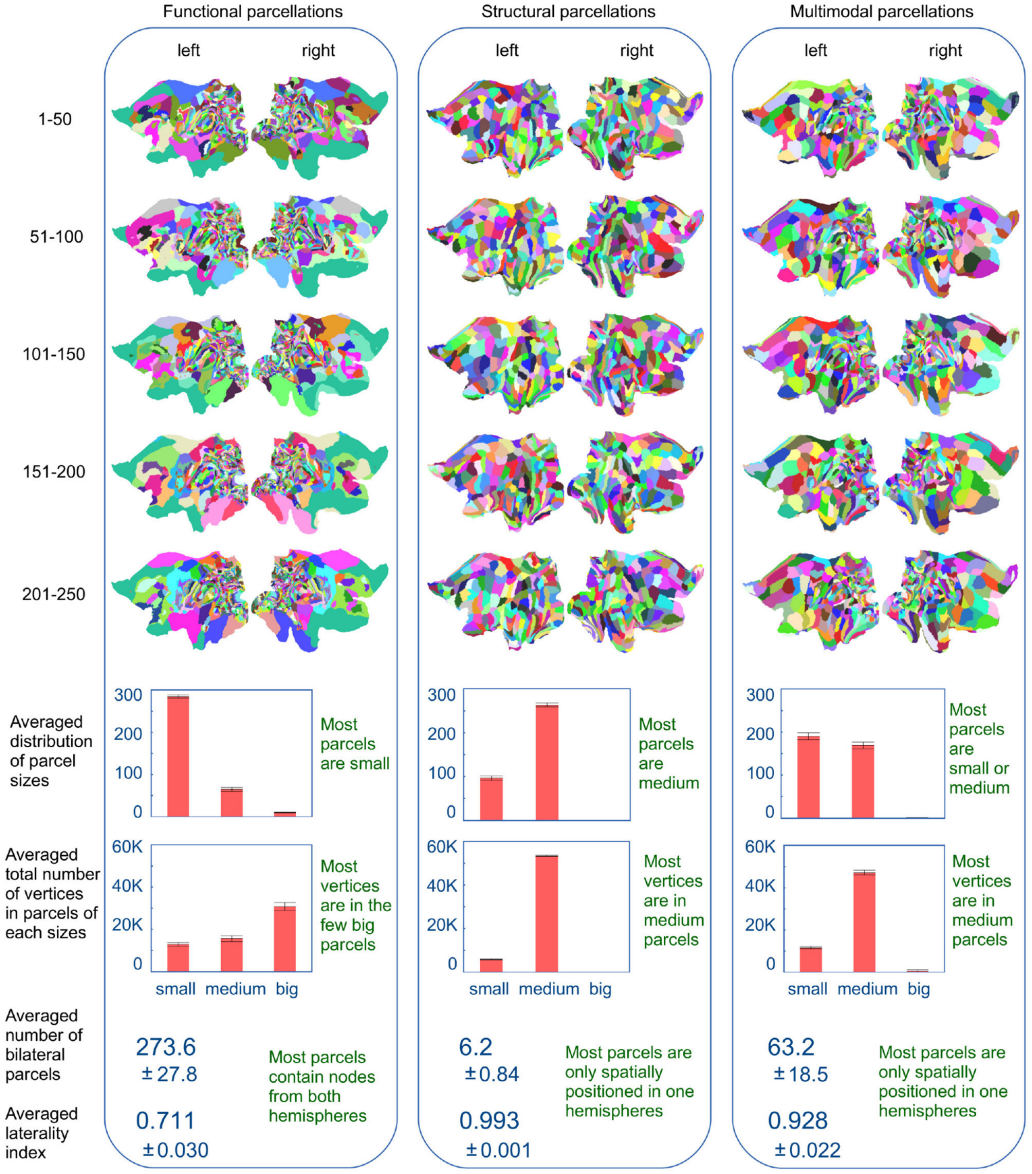
Flatmaps of the inferred parcellations for the five populations of 50 subjects, using the different modalities. Parcels are separated into three groups based on their size: small (<100 vertices), medium (between 100 and 1,000 vertices) and big (more than 1,000 vertices). Also shown, for the per-modality averages of the five parcellations, is the number of parcels that contain nodes from both hemispheres and how evenly these nodes are split across the two hemispheres. Errorbars and ± indicate the standard deviation over the five parcellations.

Figure 6 shows flatmap representations of the inferred parcellations (based on the extraction of 360 parcels as used in the HCP_MMP1.0 atlas), for each of the five 50-subject populations. There is a clear similarity between the parcellations of various training populations within each modality, while there are clear characteristic differences across modalities. The functional parcellations (left column) show a high density of small, elongated parcels seemingly located in the posterior cortex, while the majority of vertices are located in few very large parcels covering the remainder of the cortex. In contrast, the structural parcellations (middle column) show spatial compactness with the majority of clusters being of a similar size. The multimodal parcellations (right column) appear to inherit features from both modalities, showing a variance of cluster sizes and shapes. Compared to the functional parcellations, the posterior cortex is segregated into fewer parcels, though still many more than in the structural parcellations. The parcels in the rest of the cortex have also inherited the spatial compactness of the structural parcellations. Although they are slightly larger, they represent a finer segregation of the cortex. The multimodal parcellations are therefore more detailed in the posterior cortex than the structural parcellations and more detailed in the rest of the cortex than the functional parcellations.

Figure 6 (lower panel) shows histograms for the distribution of cluster sizes averaged across the parcellations for all five 50-subject populations. For the functional parcellations most parcels are very small (<100 vertices) while the majority of nodes are located in extremely large parcels (larger than 1,000 nodes). In contrast, the structural populations are homogeneous, such that the majority of nodes are located in medium sized clusters (between 100 and 1,000 parcels) which is also the most common parcel size. Once again, the multimodal parcellations seem to inherit features from both modalities. Compared to the functional populations, the cluster size distribution is shifted toward larger parcels, with the concurrent removal of the few extremely large parcels, such that the majority of vertices are now located in the medium sized parcels. Furthermore, the panel shows the extent to which parcels are common to both hemispheres. This is shown both as the number of bilateral parcels and as an index, representing how evenly the nodes of the individual parcels are split across hemispheres. This laterality index for a parcel is computed as $\frac{max\left(N_{left},N_{right}\right)}{N_{left}+N_{right}}$, where *N*_*left*_ and *N*_*right*_ is the number of nodes within the parcel, that, respectively belongs to the left and right hemisphere. An average laterality index of 0.5 would indicate that all parcels are equally split across the two hemispheres, while an average index of 1 would indicate that all parcels are unilateral. The functional parcellations are significantly more bilateral (273 parcels out of 360) than both the structural (6 parcels) and multimodal (63 parcels). The average laterality index further indicates that the individual functional parcels tend to be bilateral, whilst this is uncommon for both structural and multimodal parcels.

**Figure 7:**
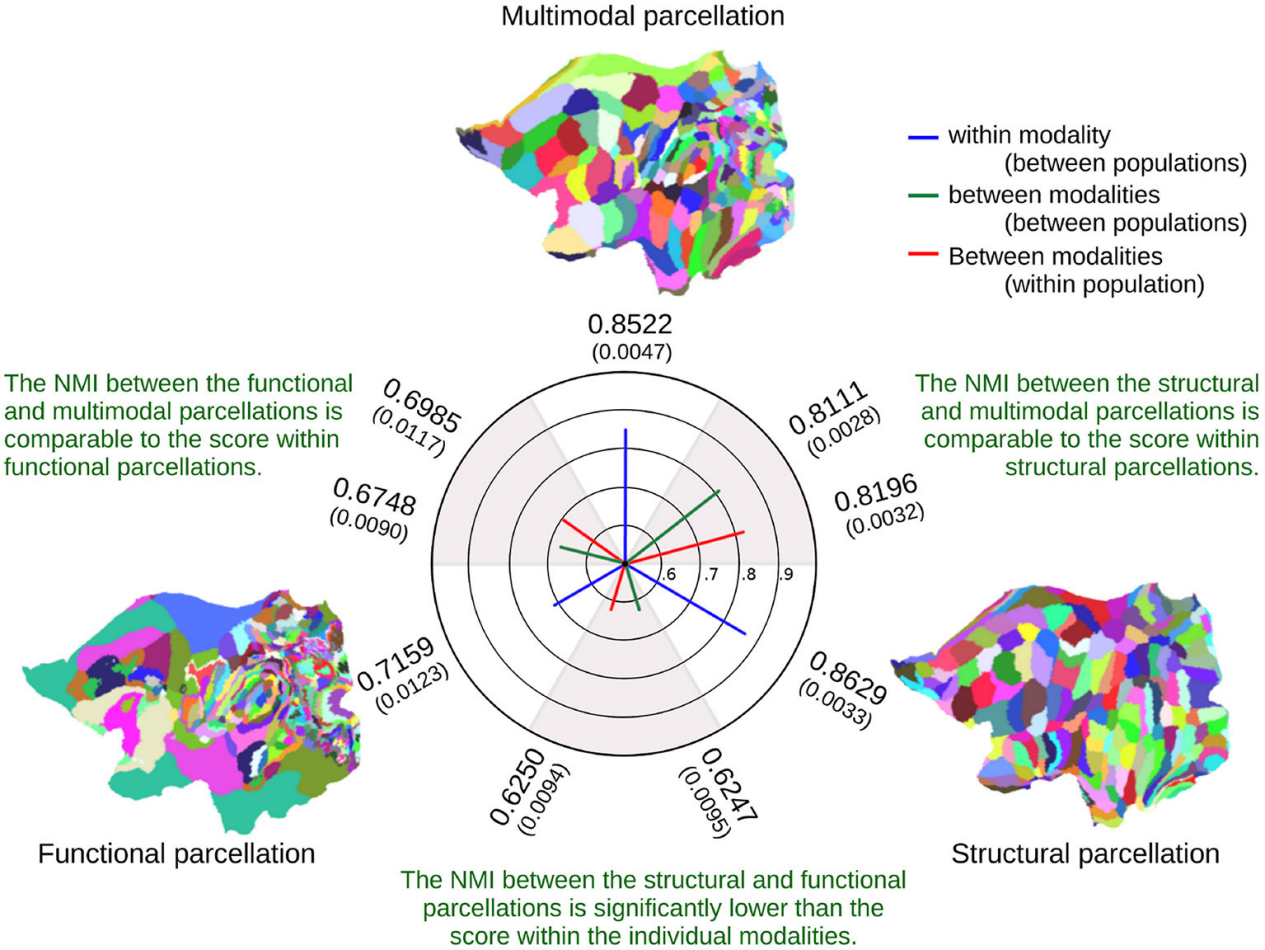
Averaged Normalized Mutual Information (NMI) for the inferred parcellations between and within modalities, showing the average NMI from 10 comparisons of parcellations between populations and five comparisons within populations. The standard deviations are shown in parenthesis. The flatmaps illustrate one of the five parcellations for a single 50-subject population, showing only the left hemisphere.

Figure 7 indicates the similarity of the inferred parcellations, as measured by Normalized Mutual Information (NMI), between and within modalities (see also section 2). Functional parcellations are inherently noisy, as evidenced by their mutual information being far lower than those of the structural parcellations. Furthermore, the multimodal parcellations are not penalized by the noise of the functional data, as they retain an NMI almost on par with the NMI within structural parcellations. The figure further indicates that the multimodal parcellations are more in agreement with the structural parcellations than with the functional, though the functional parcellations are more in agreement with the multimodal parcellation than with the structural. This implies that the multimodal model has determined a consensus to which both the functional and structural parcellations are more in agreement than they are with each other.

### 3.2. Predictive Performance

**Figure 8:**
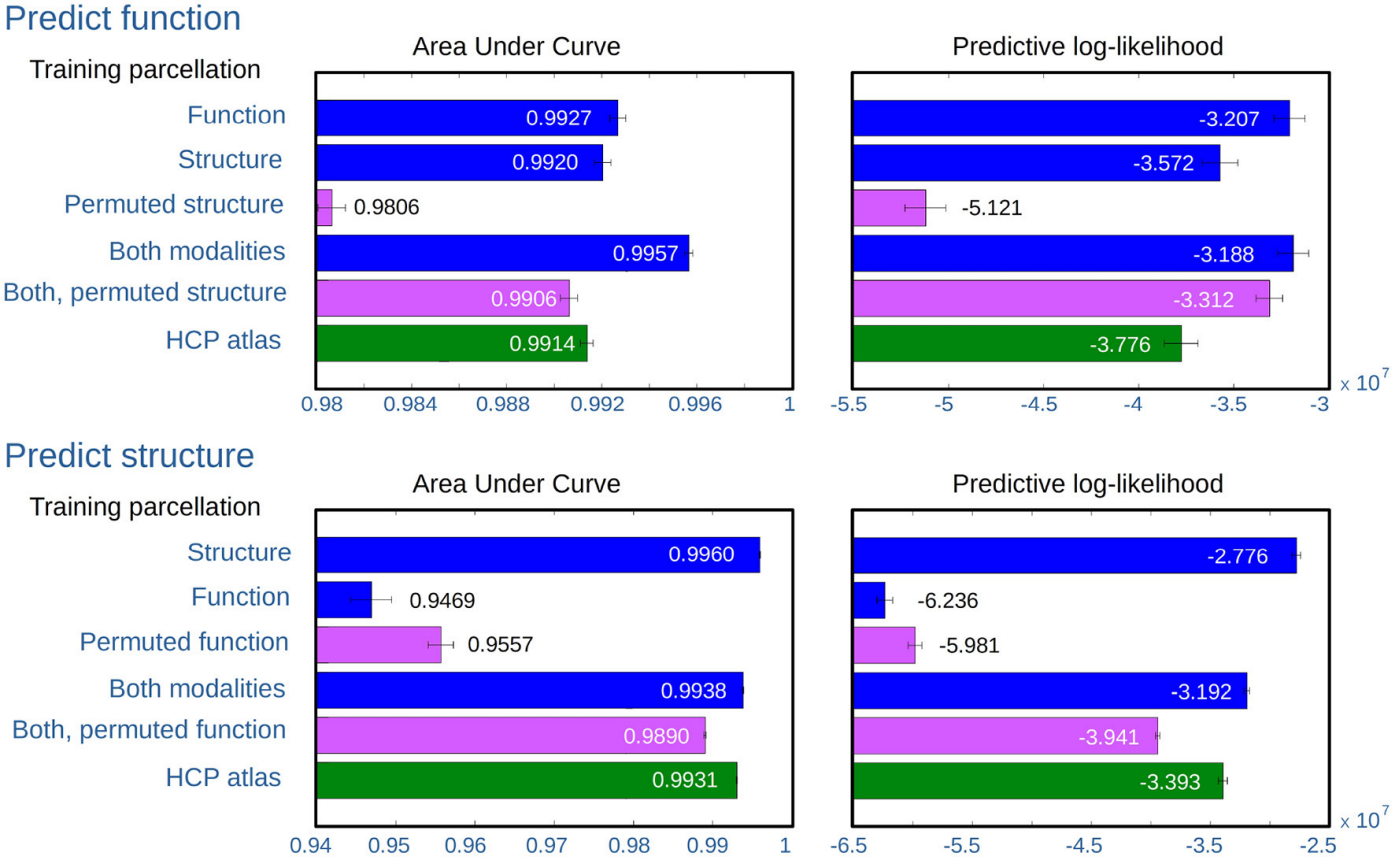
AUC and expected predictive log-likelihood when predicting structural and functional hold-out network. The bars show the average score as obtained using the following different parcellations: (1) SBM on the same modality as the test networks, (2) SBM on the other modality, (3) SBM on the permuted networks for the other modality, (4) jointly modeling both modalities, (5) jointly modeling both modalities with the network for the other modality permuted, and (6) using the parcellation defined by the HCP atlas. For each of the five training populations, four evaluations are computed for each modality configuration, when respectively predicting from the training graph to each of the other four graphs of the same modality (providing a total of 20 predictions). The mean value is shown for each bar while the whiskers indicate the standard deviation of the mean correcting by the five independently acquired networks ($\pm \mathrm{std}/\sqrt{5}$).

Figure 8 shows the results of predicting functional and structural hold-out networks using the following inferred parcellations:

- Data-driven parcellations for a single modality, inferred from either the same modality as the hold-out networks, the other modality, or the permuted version of the other modality (enforcing non-correspondence).
- Data-driven parcellations for the multimodal model, inferred from both modalities where the non-predicted modality is considered both with and without permutation (enforcing non-correspondence).
- The fixed multi-modal HCP_MMP1.0 atlas.

Both the area under curve of the receiver operator characteristic (AUC) and expected predictive likelihood scores (for details see section 2) are individually computed and averaged for the parcellations inferred for the five 50-subject populations.

Predicting both modalities, the figure shows that predicting the same modality as that from which the parcellation was inferred provides good predictions according to both predictive metrics. While structural parcellations predict function on par with the HCP atlas, functional parcellations are comparably poor predictors of structure. However, the consensus parcellations of joint modeling provide good predictions of both modalities, though slightly better for predicting function than structure.

As expected, breaking the consensus in the joint model, by using a permuted modality, decreases the predictive performance, but not to the same extent as training on the permuted data alone. In predicting functional networks, structural parcellations perform well, and far better than parcellations inferred from permuted structure. Functional parcellations on the other hand are very poor predictors of structural networks, to such a degree that parcellations inferred from the permuted functional networks actually predict better.

## 4. Discussion

Herein we have introduced a method for quantifying multimodal integration, which assumes that structure and function are independent realizations of the same underlying processing units. While a lot of effort has recently focused on developing multilayered networks of brain connectivity, data-driven quantifications of these remains challenging and mostly limited to comparisons between modalities (Koch et al., 2002; Vincent et al., 2007; Skudlarski et al., 2008; Greicius et al., 2009; Honey et al., 2009; Sporns, 2014; Becker et al., 2015; Røge et al., 2017). By representing SC and FC graphs over the same set of network nodes, the framework proposed utilizes a stochastic blockmodel (SBM) to obtain a unified parcellation while admitting modality-specific connectivity profiles. A benefit of the considered approach, integrating connectomes using the stochastic block model is that it naturally accounts for modality specific connectivity structure between the extracted units of processing while enforcing consistency across modalities in terms of the extracted parcels thereby providing a simple computationally tractable data-driven approach. As such, the SBM can be considered a data driven approach to network compression in terms of shared learned parcels ***z*** and how the connectivity structure between these parcels form modality specific networks at the level of parcels with link densities (i.e., connectivity strengths) ***η***^(*m*)^. This facilitates direct interpretation of large connectomes at the level of connectivity structure between the extracted parcels. As opposed to other connectivity based clustering approaches such as k-means, hierarchical, and spectral clustering that are generic clustering procedures designed for feature data (Eickhoff et al., 2015; Gabasova et al., 2017; Liu et al., 2020; Reuter et al., 2020) a benefit of the SBM is that it provides a statistically grounded generative model of networks. Thereby, the quantification of these unified parcellations by the SBM naturally follows the predictive framework outlined in Figure 4 that assesses the performance of predicting independent held-out test graphs as a substitute in the absence of ground truth (Albers et al., 2021).

### 4.1. Qualitative Differences in Functional, Structural, and Joint Parcellations

We observe that the characteristics of the inferred parcellations heavily depend on the modality. In particular, we observe that functional parcels are much more bilateral and have a wider distribution of unit sizes. This can be attributed to the bilateral co-activation of similar functional units across both hemispheres as also observed from seed based and independent component analysis (ICA) of the HCP resting state data (Smith et al., 2013). In contrast, structural connectivity generates much more unilateral units with no parcels having more than 1,000 voxels. We attribute the unilaterality to limitations in tractography in terms of delineating inter-hemispheric structural connections and long-distance pathways (Van Essen et al., 2014; Knösche et al., 2015; Maier-Hein et al., 2017). The joint modeling also exhibits substantial differences to both the modality-specific parcellations. As such, the identified consensus representation is reduced of modality specific biases as observed from both the size distribution and laterality index being in between the structural and functional parcellations. This joint representation possesses substantial agreement with both modalities, in so much that we find that the normalized mutual information (NMI) between the joint parcellation and the functional and structural parcellations are in higher agreement than the NMI between the functional and structural parcellations. These results demonstrate that both modalities each contribute unique information about the brain's underlying organization influencing the joint representation of parcels.

### 4.2. Statistical Evidence for Canonical Processing Units

Predicting the organization of each modality based on a data-driven parcellation of the other we found that structural parcellations predicted almost on par with functional parcellations and much better than prediction when enforcing non-correspondence. However, for structural data we found that the functional parcellation was a poor predictor to the extent that the permutation procedure, which broke correspondence yet both preserved size distribution and enforced spatial homogeneity, was a better predictor of function. We attribute this to the parcels of the permuted functional data preserving spatial homogeneity which more favorably accounts for structure than the highly bilateral parcels extracted by function as observed in Figure 2. Notably, this supports the benefit of imposing spatial constraints when modeling structural connectomes, as proposed in Baldassano et al. (2015) in which parcels are constrained to be spatially connected, and emphasizes that the modality-specific parcellations are heavily influenced by modality-specific biases. The consensus representation here provides a representation reduced of the modality-specific biases. Interestingly, we find that the predictions of the more noisy functional connectome are improved using the consensus representation, whereas the structural connectivity predictions are only mildly reduced when compared to using the modality-specific parcellations. In particular, integrating both modalities results in a consensus representation that has better predictive performance than permuting one modality thereby enforcing non-correspondence.

Our results points to modality-specific biases and differences in the representation of functional and structural units. We thus do not find a direct correspondence at the level of modality-specific processing units in the brain. However, we do find that imposing canonical processing units forms a useful, practical representation of structural and functional data in high resolution that well characterize both modalities. In particular, we observe that the noisier functional modality benefits from the integration of structural information. Whereas the structural network is expected to be constant, i.e., it is a static structure, the functional connectivity estimates derived from fMRI vary and are related to activations that only use some parts of the structural network. As such, functional connectomes derived from fMRI exhibit a high degree of inter- and intra-subject variability (Poldrack et al., 2015; Zuo et al., 2019; Albers et al., 2021) and the joint modeling with structural connectivity can help here by regularizing the extracted representation despite the variability of the fMRI source.

### 4.3. Limitations

The inter-subject alignment is based on surface morphometry, according to the standard HCP processing pipeline (Glasser et al., 2013) which could conceivably bias toward one modality more than the other. Furthermore, we arbitrarily threshold the networks at 1% density. Further studies should investigate if these findings are reproducible to change in registration methods and methods for functional and structural network construction.

We considered *K* = 360 parcels as employed by the HCP-MMP1.0 atlas (Glasser et al., 2016) enabling a direct comparison in terms of the same number of extracted units of processing. However, as we saw, the structural and functional data provide fine grained resolutions in different parts of the cortex and thus have differing preferences in terms of regions using coarse and fine grained parcels. As the joint modeling provides more data upon which parcellations can be learned it may be that better joint representations can be achieved utilizing more than the considered parcels admitting fine-grained resolutions both where it is most supported by structure and function. Future work should explore the impact of resolution employed in terms of numbers of parcels invoked and whether the joint modeling can provide support for the use of more parcels.

In this study, we attempted to generate a random control by permuting the network for one modality. This, however, is not straightforward, as it requires a permutation that preserves the spatial homogeneity, shape, and size of the parcel along with the modality specific distribution of parcels. We approximated this using the inherent vertex adjacency ordering of the HCP data format, which results in a tendency to define contiguous yet elongated parcels. Future work should develop more advanced permutation procedures that accounts for parcel shape.

The model employed herein is based on the assumption that the connectivity profiles of the two modalities are independent, yet originating from the same underlying processing units. This assumption is a simplification of the expected true underlying organization of the brain (where at least at the neuronal level we would expect a strong structure-function relationship; Innocenti et al., 2014; Andersson et al., 2020), and is generally not expected to be the case (Eickhoff et al., 2017). It is thus widely believed that the connectivity structures are related although representing substantially different time scales. As such, structural paths have been observed predictive of functional connections (Røge et al., 2017; Becker et al., 2018) which is not accounted for by the SBM. Future work should consider more advanced non-linear modeling approaches inspired by recent deep learning approaches (Banka et al., 2020; Bessadok et al., 2021) that potentially can leverage the presently considered SBM to learn such dependencies of ***η***^(*m*)^.

The model assumes that the modalities contribute equally and share similar properties in regards to resolution, spatial homogeneity, level of noise, and that both modalities are equally informative. Despite these constraints, our findings illustrate that the joint model allows for the identification of shared units that are useful in practice. However, even when multimodal integration allows for good predictions, care must be taken regarding the inference of the purported underlying organization that would account for such findings. This is because a perceived agreement between the modalities does not necessarily mean that the spatial extent of the brain regions, and the borders between them, are in fact located in the manner implied by the data-driven parcellation (Eickhoff et al., 2017).

The joint modeling of functional and structural connectivity extracts a consensus representation that can be reduced of modality specific biases. However, as we have no ground truth information in regards to the true optimal units of processing, the presented evaluation can be considered a qualitative assessment demonstrating concurrence beyond a rather simple block-permuted null model. Arguably, an average representation should be a better predictor than a representation based only on the complementary modality. In general, we expect the joint modeling to provide consensus representations superior to the representations provided by each modality when both modalities exhibit similar degrees of noise. However, if one modality is substantially less biased from the true (unknown) underlying representation the joint modeling may be driven undesirably by the more biased modality. In circumstances where a priori knowledge are available of the validity of the connectivity structures of the considered modalities the joint modeling can potentially be advanced to provide more emphasis to more accurate modalities.

In the present study, we considered the perhaps most simple approach to extract functional connectivity based on zero lag Pearson correlation (Bullmore and Sporns, 2009; Smith et al., 2011; Richiardi et al., 2013). Notably, it is unclear how functional connectivity is best quantified and several approaches exist, including mutual information (Bullmore and Sporns, 2009; Mørup et al., 2010; Smith et al., 2011), wavelet correlation (Achard et al., 2006), lagged correlation and partial correlation, as well as approaches quantifying directionality (see also Smith et al., 2011; Richiardi et al., 2013 for reviews). Herein we considered only positive correlation, while negative functional correlations arguably also relate to structure. Note also that the examined HCP fMRI data has a high temporal and spatial resolution, which might give a poorer signal-to-noise ratio than other protocols.

The quantified water diffusion by dMRI is a noisy and indirect measure of fiber-orientation, making structural connectivity inference based on dMRI inherently uncertain. In particular, the relative low image resolution of diffusion MRI often results in partial volume regions resulting in crossing fibers needing to be disentangled (Schilling et al., 2017; Ambrosen et al., 2020). Furthermore, as different tract systems contain different numbers of axons, and hence tract volumes, structural connectivity is expected to be volume-weighted toward the major tracts, also typically explored with tracers (Innocenti et al., 2014, 2017; Van Essen et al., 2014; Jbabdi et al., 2015). Herein we extracted the structural connectivity networks using probabilistic tractography (Behrens et al., 2003, 2007). However, tractography methods are known to suffer from systematic biases, such as a preference to terminate at gyral crowns (Van Essen et al., 2014; Schilling et al., 2018), issues characterizing multiple fiber directions in the face of limited data resolution (Jbabdi et al., 2015), and difficulties tracking long distance pathways (Van Essen et al., 2014).

## 5. Conclusion

The work presented herein is a novel approach for quantifying the relation between the function and structure of the brain and the integration of these in terms of processing units. Herein we considered joint network modeling of structural and functional connectivity data, however the proposed framework naturally extends to general multimodal modeling, including additional modalities.

Using high quality data from the Human Connectome Project, we find that shared canonical processing units cannot be discredited, despite the lack of observed correspondence between the modality-specific connectivity profiles. As such, we find that integrating both modalities allows for reasonable predictions of the individual modalities, as quantified by two separate predictive metrics remaining on par or better than using either the individual modalities or the HCP_MMP1.0 atlas. This finding supports that both modalities reflect different aspects of the same underlying processing units, which allows the joint model to infer a consensus that is a mild compromise between both modalities. At this point it is unclear whether the differences and similarities of the parcellations supported by structural and functional connectome are caused by systematic biases in the derived connectomes of the two modalities due to the current limitations extracting functional and structural connectivity networks. It will thus be interesting to re-apply the presented analysis framework as the quality of extracted functional and structural connectomes in the future improve.

The presented approach is likely to benefit studies of individuals, or populations where the data quality cannot match that of the HCP, as the integration of multiple modalities would overcome noise issues if these are more disruptive than modality specific biases. Furthermore, the similarity of inferred parcellations suggests that the consensus reduces modality-specific biases, and as such the consensus representation, if evaluated on a third modality, would likely better characterize that modality than each of the training modalities separately. If there exists an underlying truth of shared processing units, that truth may come closer to be recovered the more modalities are combined, and the presented framework provides a data-driven approach to achieve this.

## Data Availability Statement

Publicly available datasets were analyzed in this study. Data used in this article were taken from the Human Connectome Project and can be accessed from db.humanconnectome.org. The generated connectomes from the HCP can be provided from the authors upon request and requires acceptance of the terms of use given at: https://www.humanconnectome.org/study/hcp-young-adult/data-use-terms.

## Ethics Statement

Ethical review and approval was not required for the study on human participants in accordance with the local legislation and institutional requirements. Written informed consent for participation was not required for this study in accordance with the national legislation and the institutional requirements.

## Author Contributions

KAl and ML contributed to conceptualization, methodology, software, formal analysis, investigation, data curation, and writing of the original draft. KAm, RR, TD, and KM contributed to conceptualization, methodology, and data curation. MS contributed to conceptualization, methodology, and software. TH, KAn, HS, and LH contributed to conceptualization, and methodology. MM contributed to conceptualization, methodology, software, formal analysis, investigation, writing of the original draft, project administration, and funding acquisition. All authors reviewed and approved the submitted version.

## Funding

This project was funded by the Lundbeck Foundation, Grant Nr. R105-9813. HS holds a 5-year professorship in precision medicine at the Faculty of Health Sciences and Medicine, University of Copenhagen which is sponsored by the Lundbeck Foundation (Grant Nr. R186-2015-2138).

## Conflict of Interest

HS has received honoraria as speaker from Sanofi Genzyme, Denmark, and Novartis, Denmark, as consultant from Sanofi Genzyme, Denmark, Lophora, Denmark, and Lundbeck AS, Denmark, and as editor-in-chief (Neuroimage Clinical) and senior editor (NeuroImage) from Elsevier Publishers, Amsterdam, The Netherlands. He has received royalties as book editor from Springer Publishers, Stuttgart, Germany and from Gyldendal Publishers, Copenhagen, Denmark. The remaining authors declare that the research was conducted in the absence of any commercial or financial relationships that could be construed as a potential conflict of interest.

## Publisher's Note

All claims expressed in this article are solely those of the authors and do not necessarily represent those of their affiliated organizations, or those of the publisher, the editors and the reviewers. Any product that may be evaluated in this article, or claim that may be made by its manufacturer, is not guaranteed or endorsed by the publisher.

## References

[B1] Achard S. Salvador R. Whitcher B. Suckling J. Bullmore E. (2006). A resilient, low-frequency, small-world human brain functional network with highly connected association cortical hubs. J. Neurosci. 26, 63–72. 10.1523/JNEUROSCI.3874-05.2006 16399673 PMC6674299

[B2] Albers K. J. Ambrosen K. S. Liptrot M. G. Dyrby T. B. Schmidt M. N. Mørup M. (2021). Using connectomics for predictive assessment of brain parcellations. NeuroImage 238, 118170. 10.1016/j.neuroimage.2021.118170 34087365

[B3] Albers K. J. Moth A. L. A. Mørup M. Schmidt M. N. (2013). Large scale inference in the infinite relational model: gibbs sampling is not enough, in 2013 IEEE International Workshop on Machine Learning for Signal Processing (MLSP), 1–6. 10.1109/MLSP.2013.6661904

[B4] Ambrosen K. S. Albers K. J. Dyrby T. B. Schmidt M. N. Morup M. (2014). Nonparametric bayesian clustering of structural whole brain connectivity in full image resolution, in 2014 International Workshop on Pattern Recognition in Neuroimaging, 1–4. 10.1109/PRNI.2014.6858507

[B5] Ambrosen K. S. Eskildsen S. F. Hinne M. Krug K. Lundell H. Schmidt M. N.. (2020). Validation of structural brain connectivity networks: the impact of scanning parameters. NeuroImage 204, 116207. 10.1016/j.neuroimage.2019.116207 31539592

[B6] Ambrosen K. S. Herlau T. Dyrby T. Schmidt M. N. Mørup M. (2013). Comparing structural brain connectivity by the infinite relational model, in 2013 International Workshop on Pattern Recognition in Neuroimaging (PRNI), 50–53. 10.1109/PRNI.2013.22

[B7] Amico E. Go ni J. (2018). Mapping hybrid functional-structural connectivity traits in the human connectome. Netw. Neurosci. 2, 306–322. 10.1162/netn_a_00049 30259007 PMC6145853

[B8] Andersen K. W. Herlau T. Mørup M. Schmidt M. N. Madsen K. H. Lyksborg M.. (2012a). Joint modelling of structural and functional brain networks, in 2nd NIPS Workshop on Machine Learning and Interpretation in NeuroImaging (MLINI 2012) (Lake Tahoe).

[B9] Andersen K. W. Madsen K. H. Siebner H. R. Schmidt M. N. Mørup M. Hansen L. K. (2014). Non-parametric bayesian graph models reveal community structure in resting state fMRI. NeuroImage 100, 301–315. 10.1016/j.neuroimage.2014.05.083 24914522

[B10] Andersen K. W. Mørup M. Siebner H. Madsen K. H. Hansen L. K. (2012b). Identifying modular relations in complex brain networks, in 2012 IEEE International Workshop on Machine Learning for Signal Processing (Santander), 1–6. 10.1109/MLSP.2012.6349739

[B11] Andersson M. Kjer H. M. Rafael-Patino J. Pacureanu A. Pakkenberg B. Thiran J.-P.. (2020). Axon morphology is modulated by the local environment and impacts the noninvasive investigation of its structure-function relationship. Proc. Natl. Acad. Sci. U.S.A. 117, 33649–33659. 10.1073/pnas.2012533117 33376224 PMC7777205

[B12] Baldassano C. Beck D. M. Fei-Fei L. (2015). Parcellating connectivity in spatial maps. PeerJ 3, e784 10.7717/peerj.784 25737822 PMC4338796

[B13] Banka A. Buzi I. Rekik I. (2020). Multi-view brain hyperconnectome autoencoder for brain state classification, in International Workshop on PRedictive Intelligence in MEdicine (Lima: Springer), 101–110. 10.1007/978-3-030-59354-4_10

[B14] Battiston F. Nicosia V. Chavez M. Latora V. (2017). Multilayer motif analysis of brain networks. Chaos 27, 047404 10.1063/1.4979282 28456158

[B15] Becker C. O. Pequito S. Pappas G. J. Miller M. B. Grafton S. T. Bassett D. S.. (2015). Accurately predicting functional connectivity from diffusion imaging. arXiv preprint arXiv:1512.02602. 10.1038/s41598-017-18769-x PMC5780460 29362436

[B16] Becker C. O. Pequito S. Pappas G. J. Miller M. B. Grafton S. T. Bassett D. S.. (2018). Spectral mapping of brain functional connectivity from diffusion imaging. Sci. Rep. 8, 1–15. 10.1038/s41598-017-18769-x 29362436 PMC5780460

[B17] Behrens T. Berg H. J. Jbabdi S. Rushworth M. Woolrich M. (2007). Probabilistic diffusion tractography with multiple fibre orientations: what can we gain? Neuroimage 34, 144–155. 10.1016/j.neuroimage.2006.09.018 17070705 PMC7116582

[B18] Behrens T. Woolrich M. Jenkinson M. Johansen-Berg H. Nunes R. Clare S.. (2003). Characterization and propagation of uncertainty in diffusion-weighted MR imaging. Magn. Reson. Med. 50, 1077–1088. 10.1002/mrm.10609 14587019

[B19] Bessadok A. Mahjoub M. A. Rekik I. (2021). Graph neural networks in network neuroscience. arXiv preprint arXiv:2106.03535. 10.1109/TPAMI.2022.3209686 36155474

[B20] Betzel R. F. Bassett D. S. (2016). Multi-scale brain networks. Neuroimage 160, 73–83. 10.1016/j.neuroimage.2016.11.006 27845257 PMC5695236

[B21] Buldú J. M. Porter M. A. (2017). Frequency-based brain networks: from a multiplex framework to a full multilayer description. Netw. Neurosci. 2, 418–441. 10.1162/netn_a_00033 30294706 PMC6147638

[B22] Bullmore E. Sporns O. (2009). Complex brain networks: graph theoretical analysis of structural and functional systems. Nat. Rev. Neurosci. 10, 186–198. 10.1038/nrn2575 19190637

[B23] Chu S.-H. Parhi K. K. Lenglet C. (2018). Function-specific and enhanced brain structural connectivity mapping via joint modeling of diffusion and functional MRI. Sci. Rep. 8, 1–19. 10.1038/s41598-018-23051-9 29549287 PMC5856752

[B24] Clauset A. Moore C. Newman M. E. (2008). Hierarchical structure and the prediction of missing links in networks. Nature 453, 98–101. 10.1038/nature06830 18451861

[B25] De Domenico M. (2017). Multilayer modeling and analysis of human brain networks. Giga Sci. 6, 1–8. 10.1093/gigascience/gix004 28327916 PMC5437946

[B26] Deslauriers-Gauthier S. Lina J.-M. Butler R. Whittingstall K. Gilbert G. Bernier P.-M.. (2019). White matter information flow mapping from diffusion MRI and EEG. NeuroImage 201, 116017 10.1016/j.neuroimage.2019.116017 31319180

[B27] Diedrichsen J. Shadmehr R. (2005). Detecting and adjusting for artifacts in fMRI time series data. Neuroimage 27, 624–634. 10.1016/j.neuroimage.2005.04.039 15975828 PMC1479857

[B28] Dsouza N. S. Nebel M. B. Crocetti D. Robinson J. Mostofsky S. Venkataraman A. (2021). M-GCN: A multimodal graph convolutional network to integrate functional and structural connectomics data to predict multidimensional phenotypic characterizations, in Medical Imaging With Deep Learning (Lübeck).

[B29] Eickhoff S. B. Constable R. T. Yeo B. T. (2017). Topographic organization of the cerebral cortex and brain cartography. NeuroImage 170, 332–347. 10.1016/j.neuroimage.2017.02.018 28219775 PMC5563483

[B30] Eickhoff S. B. Thirion B. Varoquaux G. Bzdok D. (2015). Connectivity-based parcellation: critique and implications. Hum. Brain Mapp. 36, 4771–4792. 10.1002/hbm.22933 26409749 PMC6869530

[B31] Feinberg D. A. Moeller S. Smith S. M. Auerbach E. Ramanna S. Glasser M. F.. (2010). Multiplexed echo planar imaging for sub-second whole brain fmri and fast diffusion imaging. PLoS ONE 5, e15710 10.1371/journal.pone.0015710 21187930 PMC3004955

[B32] Filip A.-C. Azevedo T. Passamonti L. Toschi N. Lio P. (2020). A novel graph attention network architecture for modeling multimodal brain connectivity, in 2020 42nd Annual International Conference of the IEEE Engineering in Medicine & Biology Society (EMBC) (Montréal), 1071–1074. 10.1109/EMBC44109.2020.9176613 33018171

[B33] Fornito A. Bullmore E. T. (2012). Connectomic intermediate phenotypes for psychiatric disorders. Front. Psychiatry 3:32. 10.3389/fpsyt.2012.00032 22529823 PMC3329878

[B34] Gabasova E. Reid J. Wernisch L. (2017). Clusternomics: Integrative context-dependent clustering for heterogeneous datasets. PLoS Comput. Biol. 13, e1005781 10.1371/journal.pcbi.1005781 29036190 PMC5658176

[B35] Glasser M. F. Coalson T. S. Robinson E. C. Hacker C. D. Harwell J. Yacoub E.. (2016). A multi-modal parcellation of human cerebral cortex. Nature 536, 171–178. 10.1038/nature18933 27437579 PMC4990127

[B36] Glasser M. F. Sotiropoulos S. N. Wilson J. A. Coalson T. S. Fischl B. Andersson J. L.. (2013). The minimal preprocessing pipelines for the human connectome project. Neuroimage 80, 105–124. 10.1016/j.neuroimage.2013.04.127 23668970 PMC3720813

[B37] Gong G. He Y. Concha L. Lebel C. Gross D. W. Evans A. C.. (2009). Mapping anatomical connectivity patterns of human cerebral cortex using *in vivo* diffusion tensor imaging tractography. Cereb. Cortex 19, 524–536. 10.1093/cercor/bhn102 18567609 PMC2722790

[B38] Greicius M. D. Supekar K. Menon V. Dougherty R. F. (2009). Resting-state functional connectivity reflects structural connectivity in the default mode network. Cereb. Cortex 19, 72–78. 10.1093/cercor/bhn059 18403396 PMC2605172

[B39] Griffanti L. Salimi-Khorshidi G. Beckmann C. F. Auerbach E. J. Douaud G. Sexton C. E.. (2014). ICA-based artefact removal and accelerated fMRI acquisition for improved resting state network imaging. NeuroImage 95, 232–247. 10.1016/j.neuroimage.2014.03.034 24657355 PMC4154346

[B40] Hermundstad A. M. Bassett D. S. Brown K. S. Aminoff E. M. Clewett D. Freeman S.. (2013). Structural foundations of resting-state and task-based functional connectivity in the human brain. Proc. Natl. Acad. Sci. U.S.A. 110, 6169–6174. 10.1073/pnas.1219562110 23530246 PMC3625268

[B41] Hinne M. Ambrogioni L. Janssen R. J. Heskes T. van Gerven M. A. (2014). Structurally-informed bayesian functional connectivity analysis. NeuroImage 86, 294–305. 10.1016/j.neuroimage.2013.09.075 24121202

[B42] Honey C. Sporns O. Cammoun L. Gigandet X. Thiran J.-P. Meuli R.. (2009). Predicting human resting-state functional connectivity from structural connectivity. Proc. Natl. Acad. Sci. U.S.A. 106, 2035–2040. 10.1073/pnas.0811168106 19188601 PMC2634800

[B43] Innocenti G. M. Dyrby T. B. Andersen K. W. Rouiller E. M. Caminiti R. (2017). The crossed projection to the striatum in two species of monkey and in humans: behavioral and evolutionary significance. Cereb. Cortex 27, 3217–3230. 10.1093/cercor/bhw161 27282154

[B44] Innocenti G. M. Vercelli A. Caminiti R. (2014). The diameter of cortical axons depends both on the area of origin and target. Cereb. Cortex 24, 2178–2188. 10.1093/cercor/bht070 23529006

[B45] Jbabdi S. Sotiropoulos S. N. Haber S. N. Van Essen D. C. Behrens T. E. (2015). Measuring macroscopic brain connections *in vivo. Nat. Neurosci*. 18, 1546 10.1038/nn.4134 26505566

[B46] Jbabdi S. Sotiropoulos S. N. Savio A. M. Gra na M. Behrens T. E. (2012). Model-based analysis of multishell diffusion MR data for tractography: how to get over fitting problems. Magn. Reson. Med. 68, 1846–1855. 10.1002/mrm.24204 22334356 PMC3359399

[B47] Kaiser M. (2013). The potential of the human connectome as a biomarker of brain disease. Front. Hum. Neurosci. 7, 484 10.3389/fnhum.2013.00484 23966935 PMC3744009

[B48] Kemp C. Tenenbaum J. B. Griffiths T. L. Yamada T. Ueda N. (2006). Learning systems of concepts with an infinite relational model, in AAAI, Vol. 3, 5 (Boston, MA).

[B49] Kim S.-G. Bandettini P. A. (2012). Principles of BOLD Functional MRI. Boston, MA: Springer US, 293–303. 10.1007/978-1-4419-0345-7_16

[B50] Knösche T. R. Anwander A. Liptrot M. Dyrby T. B. (2015). Validation of tractography: comparison with manganese tracing. Hum. Brain Mapp. 36, 4116–4134. 10.1002/hbm.22902 26178765 PMC5034837

[B51] Koch M. A. Norris D. G. Hund-Georgiadis M. (2002). An investigation of functional and anatomical connectivity using magnetic resonance imaging. Neuroimage 16, 241–250. 10.1006/nimg.2001.1052 11969331

[B52] Li Y. Mateos G. Zhang Z. (2021). Learning to model the relationship between brain structural and functional connectomes. arXiv preprint arXiv:2112.09906. 10.48550/arXiv.2112.09906

[B53] Liu X. Eickhoff S. B. Hoffstaedter F. Genon S. Caspers S. Reetz K.. (2020). Joint multi-modal parcellation of the human striatum: functions and clinical relevance. Neurosci. Bull. 36, 1123–1136. 10.1007/s12264-020-00543-1 32700142 PMC7532244

[B54] Maier-Hein K. H. Neher P. F. Houde J.-C. Côté M.-A. Garyfallidis E. Zhong J.. (2017). The challenge of mapping the human connectome based on diffusion tractography. Nat. Commun. 8, 1–13. 10.1038/s41467-017-01285-x 29116093 PMC5677006

[B55] Moeller S. Yacoub E. Olman C. A. Auerbach E. Strupp J. Harel N.. (2010). Multiband multislice GE-EPI at 7 tesla, with 16-fold acceleration using partial parallel imaging with application to high spatial and temporal whole-brain fMRI. Magn. Reson. Med. 63, 1144–1153. 10.1002/mrm.22361 20432285 PMC2906244

[B56] Mørup M. Madsen K. Dogonowski A.-M. Siebner H. Hansen L. K. (2010). Infinite relational modeling of functional connectivity in resting state fMRI, in Advances in Neural Information Processing Systems (Vancouver, BC), 1750–1758.

[B57] Nowicki K. Snijders T. A. B. (2001). Estimation and prediction for stochastic blockstructures. J. Am. Stat. Assoc. 96, 1077–1087. 10.1198/016214501753208735

[B58] Ogawa S. Lee T.-M. Kay A. R. Tank D. W. (1990). Brain magnetic resonance imaging with contrast dependent on blood oxygenation. Proc. Natl. Acad. Sci. U.S.A. 87, 9868–9872. 10.1073/pnas.87.24.9868 2124706 PMC55275

[B59] Poldrack R. A. Laumann T. O. Koyejo O. Gregory B. Hover A. Chen M.-Y.. (2015). Long-term neural and physiological phenotyping of a single human. Nat. Commun. 6, 1–15. 10.1038/ncomms9885 26648521 PMC4682164

[B60] Reuter N. Genon S. Masouleh S. K. Hoffstaedter F. Liu X. Kalenscher T.. (2020). Cbptools: a python package for regional connectivity-based parcellation. Brain Struct. Funct. 225, 1261–1275. 10.1007/s00429-020-02046-1 32144496 PMC7271019

[B61] Richiardi J. Achard S. Bunke H. Van De Ville D. (2013). Machine learning with brain graphs: predictive modeling approaches for functional imaging in systems neuroscience. IEEE Signal Process. Mag. 30, 58–70. 10.1109/MSP.2012.2233865

[B62] Røge R. Ambrosen K. S. Albers K. J. Eriksen C. T. Liptrot M. G. Schmidt M. N.. (2017). Whole brain functional connectivity predicted by indirect structural connections, in 2017 International Workshop on Pattern Recognition in Neuroimaging (PRNI) (Toronto, ON), 1–4. 10.1109/PRNI.2017.7981496

[B63] Salimi-Khorshidi G. Douaud G. Beckmann C. F. Glasser M. F. Griffanti L. Smith S. M. (2014). Automatic denoising of functional MRI data: combining independent component analysis and hierarchical fusion of classifiers. NeuroImage 90, 449–468. 10.1016/j.neuroimage.2013.11.046 24389422 PMC4019210

[B64] Schilling K. Gao Y. Janve V. Stepniewska I. Landman B. A. Anderson A. W. (2017). Can increased spatial resolution solve the crossing fiber problem for diffusion MRI? NMR Biomed. 30, e3787 10.1002/nbm.3787 28915311 PMC5685916

[B65] Schilling K. Gao Y. Janve V. Stepniewska I. Landman B. A. Anderson A. W. (2018). Confirmation of a gyral bias in diffusion MRI fiber tractography. Hum. Brain Mapp. 39, 1449–1466. 10.1002/hbm.23936 29266522 PMC5807146

[B66] Schmidt M. N. Mørup M. (2013). Nonparametric Bayesian modeling of complex networks: an introduction. Signal Process. Mag. 30, 110–128. 10.1109/MSP.2012.2235191

[B67] Setsompop K. Gagoski B. A. Polimeni J. R. Witzel T. Wedeen V. J. Wald L. L. (2012). Blipped-controlled aliasing in parallel imaging for simultaneous multislice echo planar imaging with reduced G-factor penalty. Magn. Reson. Med. 67, 1210–1224. 10.1002/mrm.23097 21858868 PMC3323676

[B68] Skudlarski P. Jagannathan K. Calhoun V. D. Hampson M. Skudlarska B. A. Pearlson G. (2008). Measuring brain connectivity: diffusion tensor imaging validates resting state temporal correlations. Neuroimage 43, 554–561. 10.1016/j.neuroimage.2008.07.063 18771736 PMC4361080

[B69] Smith S. M. Beckmann C. F. Andersson J. Auerbach E. J. Bijsterbosch J. Douaud G.. (2013). Resting-state fMRI in the human connectome project. Neuroimage 80, 144–168. 10.1016/j.neuroimage.2013.05.039 23702415 PMC3720828

[B70] Smith S. M. Miller K. L. Salimi-Khorshidi G. Webster M. Beckmann C. F. Nichols T. E.. (2011). Network modelling methods for fMRI. Neuroimage 54, 875–891. 10.1016/j.neuroimage.2010.08.063 20817103

[B71] Sporns O. (2014). Contributions and challenges for network models in cognitive neuroscience. Nat. Neurosci. 17, 652–660. 10.1038/nn.3690 24686784

[B72] Tononi G. Sporns O. Edelman G. M. (1994). A measure for brain complexity: relating functional segregation and integration in the nervous system. Proc. Natl. Acad. Sci. U.S.A. 91, 5033–5037. 10.1073/pnas.91.11.5033 8197179 PMC43925

[B73] Tost H. Bilek E. Meyer-Lindenberg A. (2012). Brain connectivity in psychiatric imaging genetics. Neuroimage 62, 2250–2260. 10.1016/j.neuroimage.2011.11.007 22100419

[B74] Tzourio-Mazoyer N. Landeau B. Papathanassiou D. Crivello F. Etard O. Delcroix N.. (2002). Automated anatomical labeling of activations in SPM using a macroscopic anatomical parcellation of the MNI MRI single-subject brain. NeuroImage 15, 273–289. 10.1006/nimg.2001.0978 11771995

[B75] Vaiana M. Muldoon S. F. (2020). Multilayer brain networks. J. Nonlin. Sci. 30, 2147–2169. 10.1007/s00332-017-9436-8

[B76] van Dellen E. Hillebrand A. Douw L. Heimans J. J. Reijneveld J. C. Stam C. J. (2013). Local polymorphic delta activity in cortical lesions causes global decreases in functional connectivity. Neuroimage 83, 524–532. 10.1016/j.neuroimage.2013.06.009 23769919

[B77] Van Den Heuvel M. P. Pol H. E. H. (2010). Exploring the brain network: a review on resting-state fmri functional connectivity. Eur. Neuropsychopharmacol. 20, 519–534. 10.1016/j.euroneuro.2010.03.008 20471808

[B78] Van Essen D. C. Jbabdi S. Sotiropoulos S. N. Chen C. Dikranian K. Coalson T.. (2014). Mapping connections in humans and non-human primates: aspirations and challenges for diffusion imaging, in Diffusion MRI, 2nd Edn, eds Johansen-Berg H. Behrens T. E. J. (San Diego, CA: Elsevier), 337–358. 10.1016/B978-0-12-396460-1.00016-0

[B79] Van Essen D. C. Smith S. M. Barch D. M. Behrens T. E. Yacoub E. Ugurbil K.. (2013). The wu-minn human connectome project: an overview. Neuroimage 80, 62–79. 10.1016/j.neuroimage.2013.05.041 23684880 PMC3724347

[B80] Vega Pons S. Olivetti E. Avesani P. Dodero L. Gozzi A. Bifone A. (2016). Differential effects of brain disorders on structural and functional connectivity. Front. Neurosci. 10, 605 10.3389/fnins.2016.00605 28119556 PMC5221415

[B81] Vincent J. L. Patel G. H. Fox M. D. Snyder A. Z. Baker J. T. Van Essen D. C.. (2007). Intrinsic functional architecture in the anaesthetized monkey brain. Nature 447, 83–86. 10.1038/nature05758 17476267

[B82] Xu J. Moeller S. Strupp J. Auerbach E. Chen L. Feinberg D.. (2012). Highly accelerated whole brain imaging using aligned-blipped-controlled-aliasing multiband EPI, in Proceedings of the 20th Annual Meeting of ISMRM, Vol. 2306 (Melbourne, VIC).

[B83] Xu Z. Tresp V. Yu K. Kriegel H.-P.. (2006). Learning infinite hidden relational models, in Uncertainity in Artificial Intelligence (UAI2006) (Cambridge), 2.

[B84] Zhang S. He Z. Du L. Zhang Y. Yu S. Wang R.. (2021). Joint analysis of functional and structural connectomes between preterm and term infant brains via canonical correlation analysis with locality preserving projection. Front. Neurosci. 15, 724391 10.3389/fnins.2021.724391 34690672 PMC8526737

[B85] Zhu S. Yu K. Gong Y. (2008). Stochastic relational models for large-scale dyadic data using MCMC, in Advances in Neural Information Processing Systems (Vancouver, BC), 1993–2000. Available online at: https://papers.nips.cc/paper/2008/file/2291d2ec3b3048d1a6f86c2c4591b7e0-Paper.pdf

[B86] Zuo X.-N. Biswal B. B. Poldrack R. A. (2019). Editorial: Reliability and reproducibility in functional connectomics. Front. Neurosci. 13, 117 10.3389/fnins.2019.00117 30842722 PMC6391345

